# 

**Published:** 1870-12

**Authors:** 


*CONTENTS:*

ORIGINAL COMMUNICATIONS:

ART. I. Injury of the Head—Abscess, with absence of Symptoms, etc. By C. T. Parkes, M.D. ...... 705

ART. II. A Case of Traumatic Tetanus, treated with Hyd. Chloral. By A. H. Kinnear, M.D........... 709

ART. III. Read to the Muskingum Co. Med. Society. By A. Ball, M.D. 713

ART. IV. Clinical Experiences in Private Practice, etc. By Z. C. McElroy, M.D. ..................... 716

ART. V. A Day amongst the Surgeons of Philadelphia. By John E. Owens, M.D.................... 725

ART. VI. Prof. Beale and his Doctrines. By I. N. Danforth, M.D... 732

ART. VII. Case of Irregular Innervation. By Walter Hay, M.D..... 741

ART. VIII. Cases from the Surgical Clinic of Prof. Moses Gunn, of Rush Med. College. Reported by H. F. Chesbrough, M.D........ ... 743

SELECTIONS:
Suspension in Spinal Affections.... 745

EDITORS' BOOK TABLE................. 754
Handbook of Med. Microscopy; Pathology and Treatment of Venereal Diseases; Practical Anatomy; Treatise on Physiology and Hygiene, etc.

PAMPHLET NOTICES.................... 757

EDITORIAL.............................. 760

MEDICAL ITEMS, NEWS, AND GOSSIP... 763

LOOT.................................. 766

